# Comparison of Dietary Behaviors and the Prevalence of Metabolic Syndrome in Single- and Multi-Person Households among Korean Adults

**DOI:** 10.3390/healthcare9091116

**Published:** 2021-08-27

**Authors:** Kyung Won Lee, Dayeon Shin

**Affiliations:** 1 Department of Home Economics Education, Korea National University of Education, Cheongju 28173, Korea; kwlee@knue.ac.kr; 2 Department of Food and Nutrition, Inha University, Incheon 22212, Korea

**Keywords:** single-person households, household types, Korean adults, metabolic syndrome, Korea National Health and Nutrition Examination Survey

## Abstract

Changes in household dynamics in Korea, such as the transition from multi- to single-person households, have led to changes in individuals’ dietary behavior patterns and health status. Thus, this study aimed to compare dietary behaviors and determine the prevalence of metabolic syndrome (MetS) as well as explore factors associated with MetS according to household type among Korean adults. Using data from the Korea National Health and Nutrition Examination Survey 2014–2018, we included 21,944 Korean adults with available health examination and dietary recall data. Based on self-reported information, individuals were divided into two household types: single- and multi-person households. We used multivariable logistic regression to estimate the adjusted odds ratios (AORs) and 95% confidence intervals (CIs) for MetS and its components after adjusting for potential covariates. Among the study population, 9.19% and 90.81% lived in single-person and multi-person households, respectively. Individuals in single-person households had a higher energy intake overall and a greater percentage of energy from animal protein; total, saturated, and monounsaturated fats; and animal source foods and a lower percentage of energy from carbohydrates, plant protein, and plant source foods than those from multi-person households (all, *p* < 0.05). Individuals living in single-person rather than multi-person households were more likely to consume milk/dairy products, alcoholic and non-alcoholic beverages, oils/fats, and others but were less likely to consume vegetables/mushrooms, fruits, seaweeds, and fish/shellfish (all, *p* < 0.05). Living alone was associated with higher energy intake from main meals and foods prepared away from home but a lower dietary variety score and fewer total main meals consumed (all, *p* < 0.05). Skipping breakfast, frequent eating out, food insecurity, and MetS combination phenotypes significantly differed by household type. Individuals living alone had higher odds of MetS (AOR: 1.14, 95% CI: 1.02–1.29), abdominal obesity (AOR: 1.14, 95% CI: 1.01–1.28), elevated blood pressure (AOR: 1.28, 95% CI: 1.12–1.47), and elevated fasting blood glucose (AOR: 1.18, 95% CI: 1.05–1.33) than those living with others. Compared with those in multi-person households, individuals in single-person households tend to have health and dietary behaviors that increase vulnerability to MetS; therefore, establishing health care strategies and nutrition policies according to household type is necessary.

## 1. Introduction

According to a recent report from Statistics Korea, the number of single-person households nationwide in 2019 was approximately 5.99 million, accounting for 29.8% of all household types [1]. From 1990 to 2005, four-person households were the most common, comprising couples and their two children [2]; however, since 2010, single-person households have become the most common household type in Korea, and the proportion of single-person households is projected to increase to 37.3% by 2045 [3]. This increase has been attributed to various socioeconomic conditions, including weakening family values, deepening individualism, more unmarried individuals, job insecurity, and a worsening financial situation [4]. Living alone is now a common social phenomenon.

Changes in household dynamics, such as the transition from multi- to single-person households, have led to changes in lifestyle patterns and health status [5]. In a study of Korean elderly individuals aged ≥60 years, those living alone were approximately twice as likely to have a poor subjective health status than those living with family members [5]. Compared with those in multi-person households, those in single-person households experience loneliness, sadness, feelings of futility [6], and lower life satisfaction [7] more frequently and show an increased risk of suicidal ideation [8], indicating that single-person households are more vulnerable in terms of physical and mental health deterioration and require careful management. In addition, people living in single-person households are more likely to engage in unhealthy behaviors than those in multi-person households, showing higher rates of current smoking, drinking [9], and engaging in health-risk behaviors [10].

Metabolic syndrome (MetS) is characterized by the clustering of various risk factors for cardiovascular diseases and type 2 diabetes [11]. MetS is one of the leading causes of death among Koreans [12], and its prevalence has steadily increased over the past few decades, from 21.6% in 2007 to 22.9% in 2018, with a particularly rapid increase in men (22.5% in 2007 vs. 27.9% in 2018) [13]. Previous meta-analyses of prospective cohort studies concluded that MetS is associated with an increase not only in the incidence of cardiovascular diseases, such as stroke and myocardial infarction, but also all-cause and cardiovascular mortality [14,15,16].

Recognizing the importance of MetS in the prevalence of cardiovascular diseases and the associated mortality rate, significant efforts have been made to identify the risk factors for MetS. Insufficient physical activity, drinking, and smoking are well-known modifiable risk factors for MetS [17,18]. Sedentary lifestyles, such as frequent sitting and lying down, low levels of physical activity, and excessive screen time are also associated with an increased risk of MetS [19,20,21]. In addition, dietary factors that increase or decrease the risk of MetS have been documented. While high-density energy intake [22], refined carbohydrates [23], and sugar-sweetened beverages [24] are positively associated with higher odds of developing MetS and its components, diets rich in dietary fiber [25], antioxidants [26], and polyunsaturated fatty acids [27] play a favorable role against the development of MetS. A recent review by Hernandez-Rodas et al. also highlighted that lifestyle interventions, including modifying unhealthy dietary habits and behaviors, are important factors in the management and treatment of non-alcoholic fatty liver diseases that are related to diabetes, hypertension, and dyslipidemia [28].

However, despite the rapid change in household types, only a few studies have explored the differences in dietary behaviors between single- and multi-person households and their association with the prevalence of metabolic diseases [29,30,31,32]. Moreover, previous studies have been conducted on individuals of a particular age and sex and in a particular geographic area, making it difficult to comprehensively understand the risk factors for metabolic diseases across single- and multi-person households in Korea. Therefore, it is necessary to identify and compare the differences in health and dietary behaviors by household type in the general Korean population to reduce the social, economic, and personal burden caused by MetS.

To address these gaps in the literature, in this study, we aimed to compare the nutrient and food intake, diet quality, and dietary behaviors between Korean adults living in single- and multi-person households. We also investigated the prevalence of MetS and its individual components and the MetS combination phenotypes by household type using representative data of Korean adults.

## 2. Materials and Methods

### 2.1. Data Source and Study Population

**Figure 1:**
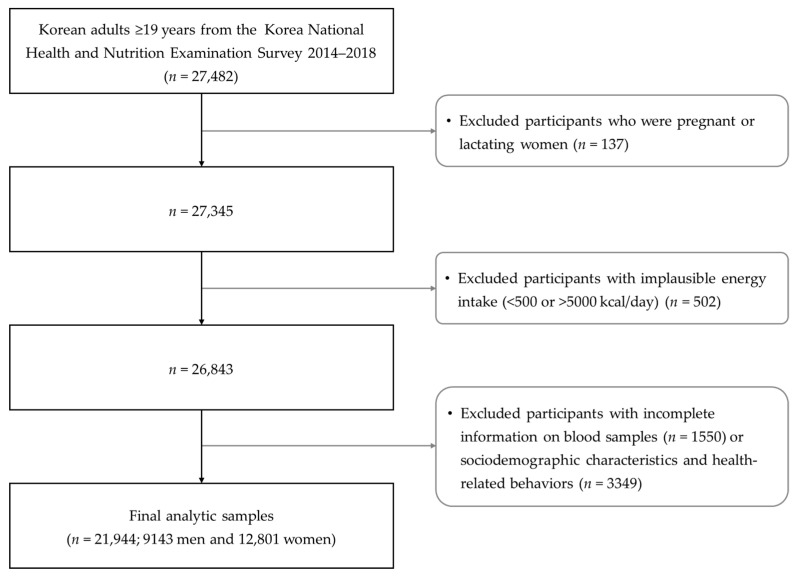
Flowchart of the study population.

We used data from the nationally representative Korea National Health and Nutrition Examination Survey (KNHANES) 2014–2018. The KNHANES is an ongoing surveillance system that collects information on several variables using a health interview, health examination, and nutrition survey to assess health and nutritional status and to monitor the prevalence of chronic diseases in Korea [33]. In this study, we included 27,482 Korean adults aged ≥19 years who participated in the KNHANES 2014–2018. We excluded those who were pregnant or lactating women (*n* = 137), had implausible energy intake (<500 or >5000 kcal/day; *n* = 502), incomplete data on laboratory (*n* = 1550), sociodemographic characteristics or health-related behaviors (*n* = 3349). Finally, 21,944 participants (9143 men and 12,801 women) were included in the current analysis (Figure 1). All study protocols and procedures of the KNHANES were reviewed and approved by the Korea Centers for Disease Control and Prevention Institutional Review Board (IRB No: 2018-01-03-P-A), and written informed consent was obtained from all study participants for each KNHANES survey [34].

### 2.2. Household Types

Participants who answered “single/one” to the question “How many people live in your household?” or who answered “single-person households” to the question “Which of the following best describes your household type?” were defined as living in single-person households.

### 2.3. Nutrient Intake and Dietary Behaviors

To understand the nutrient intake and dietary behaviors of the study participants, 24-h dietary recall data from the KNHANES were used. During the 24-h dietary recall survey, a trained interviewer collects information on the types and amounts of all food and beverage items consumed in the preceding 24 h [33]. In addition, participants were asked to report a time when and where each food and beverage item was prepared or consumed. Based on the available information, the daily total energy intake and the energy intake from plant and animal sources, carbohydrates, proteins (total, animal, and plant), and fats (total, saturated, monounsaturated, and polyunsaturated), and the prevalence of meeting or not meeting the acceptable macronutrient distribution range (AMDR) for macronutrients were computed. To compare food groups consumed and dietary variety by type of household, all food and beverage items were aggregated into 15 main food groups based on the food-grouping scheme used in the KNHANES [34] and in previous studies [35,36]. We also derived the total number of eating sessions, including main meals (breakfast, lunch, and dinner) and snacks; energy from foods prepared at home or away from home; skipping breakfast, eating all main meals alone, and eating out at least 1 time per week; and household food insecurity, to compare the dietary behaviors of individuals living in single- and multi-person households.

### 2.4. Definition of MetS and Its Components

MetS was defined as having at least three of the following conditions, based on the criteria of the National Cholesterol Education Program Adult Treatment Panel III [37], International Diabetes Federation [38]: (1) excessive waist circumference (WC) (WC ≥ 90 cm in men and ≥85 cm in women); (2) elevated triglycerides (TG) (fasting TG ≥ 150 mg/dL or receiving treatment for hypertriglyceridemia); (3) elevated blood pressure (BP) (systolic BP ≥ 130 mmHg or diastolic BP ≥ 85 mmHg or receiving treatment for or previously diagnosed with hypertension); (4) elevated fasting blood glucose (FBG) (FBG ≥ 100 mg/dL or receiving treatment for or previously diagnosed with type 2 diabetes mellitus); and (5) low high-density lipoprotein cholesterol (HDL-C) (fasting HDL-C < 40 mg/dL in men and <50 mg/dL in women).

### 2.5. Statistical Analyses

All data were analyzed using SAS version 9.4 (SAS Institute Inc., Cary, NC, USA). To account for the complex survey design of the KNHANES data, we used the PROC SURVEY procedure with the sample weights, strata, and primary sampling units recommended by the KNHANES analytic guidelines [34]. All tests were two-sided, and a *p*-value of <0.05 was considered statistically significant for all analyses.

General characteristics of the study participants according to household type were depicted using the chi-square test for categorical variables and multiple linear regressions for continuous variables. Categorical variables are expressed as numbers (weighted percentages), and continuous variables are presented as means ± standard errors. Multiple logistic regression analyses were also used to estimate odds ratios (ORs) and 95% confidence intervals (CIs) of MetS and its components according to household type (single- vs. multi-person households). We adjusted for the following covariates in the analytic models: sex (men or women), age (years), education level (≤middle school, high school, or ≥college), household income (lowest, lower middle, upper middle, and highest), marital status (married or single), occupation (unemployed or employed), region (urban or rural), drinking status (never/rarely, ≤1 time/month, or >1 time/month), smoking status (never, former smoker, or current smoker), and regular physical activity (yes or no).

## 3. Results

The general characteristics of the study population according to household type are shown in Table 1. Of the 21,944 Korean adults from the KNHANES 2014–2018, 9.19% (*n* = 2522) and 90.81% (*n* = 19,422) lived in single- and multi-person households, respectively. The mean size of the multi-person households was 3.30. Sex, age, education level, household income, marital status, occupation, region, and smoking status were significant independent correlates of household type (all, *p* < 0.05). Individuals in single-person households were more likely to be men, older, single, unemployed, and current smokers. Individuals living in single-person households were also more likely to have lower education and income levels and to reside in rural areas.

**Figure 2:**
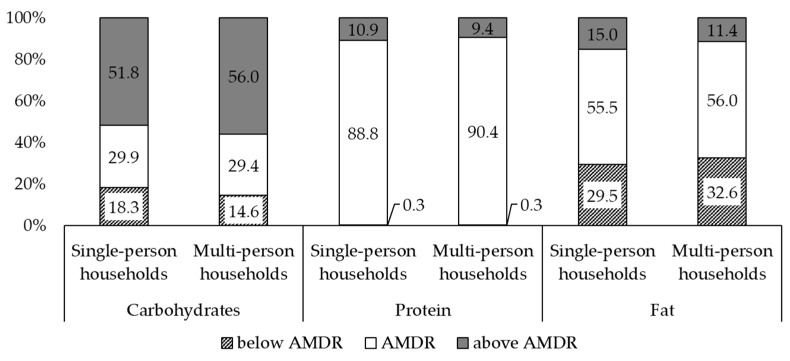
Distribution of Korean adults’ macronutrient intake according to household type, KNHANES 2014–2018. KNHANES, Korea National Health and Nutrition Examination Survey; AMDR, acceptable macronutrient distribution range. AMDR for carbohydrates, proteins, and fats were 55–65%, 7–20%, and 15–30%, respectively.

Daily energy and macronutrient intake according to household type are shown in Table 2. Individuals living in single-person households showed higher energy intake and percentage of energy from animal sources and a lower percentage of energy from plant sources than those living in multi-person households (*p* < 0.05). In terms of macronutrient intake, individuals living alone consumed more energy from animal proteins and from total, saturated, and monounsaturated fats and consumed less energy from carbohydrates and plant proteins (all, *p* < 0.05). The distribution of Korean adults’ macronutrient intake according to household type is presented in Figure 2. For carbohydrates, the proportion of participants below the AMDR was significantly higher in single-person households, while the proportion of those above the AMDR was significantly higher in multi-person households. In contrast, the proportion of inadequate fat intake was higher in multi-person households, while the proportion of excessive fat intake was higher in single-person households. There were no differences in the protein intake distribution between single- and multi-person households.

The food group consumption and dietary variety according to household type are shown in Table 3. Compared with individuals living in multi-person households, those living alone were more likely to consume milk/dairy products, alcoholic and non-alcoholic beverages, oils/fats, and others and were less likely to have vegetables/mushrooms, fruits, seaweeds, and fish/shellfish (all, *p* < 0.05). Dietary quality, measured using dietary variety scores, was significantly higher for individuals living in multi-person households than those living in single-person households, indicating that living alone was associated with greater difficulty of ensuring variety in their diet (dietary variety scores: 10.10 for single-person households vs. 10.43 for multi-person households, *p* < 0.01).

The differences in dietary behaviors between individuals living in single- versus multi-person households are presented in Table 4. There was no difference in the number of total eating and snacking episodes by household type, though those living in single-person households had fewer total main meal episodes than those living in multi-person households (*p* < 0.01). The percentage of energy from meal preparation locations differed according to the type of household. People living with others consumed more energy from foods prepared at home, while those living alone consumed more energy from foods prepared outside, including restaurants, institutional cafeterias, instant and ready-to-eat foods, and other types of foods purchased outside the home. In addition, people living alone were less likely to report eating all three main meals and were more likely to skip breakfast, consume all main meals alone, frequently eat out, and experience mild/moderate or severe food insecurity (all, *p* < 0.01).

**Figure 3:**
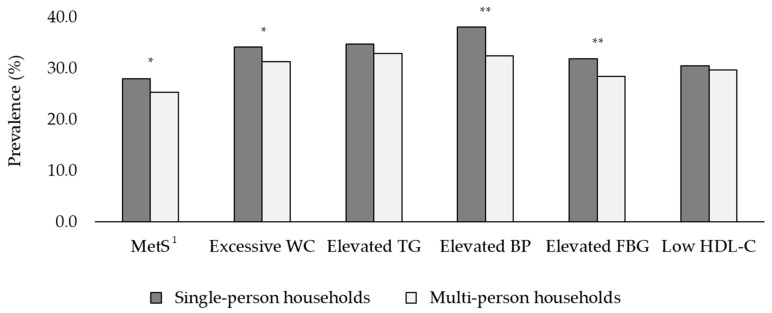
Prevalence of metabolic syndrome and its components among Korean adults according to household type, KNHANES 2014–2018. KNHANES, Korea National Health and Nutrition Examination Survey; MetS, metabolic syndrome; WC, waist circumference; TG, triglycerides; BP, blood pressure; FBG, fasting blood glucose; HDL-C, high-density lipoprotein cholesterol. Multiple logistic regression analyses were performed to estimate the prevalence of MetS and its components after adjusting for sex, age, education level, household income, marital status, occupation, region, drinking status, smoking status, and regular physical activity (* *p* < 0.05, ** *p* < 0.01). ^1^ MetS was defined as the presence of at least three of the following conditions: (1) excessive WC, (2) elevated TG, (3) elevated BP, (4) elevated FBG, and (5) low HDL-C.

The prevalence of MetS and its components according to household type is shown in Figure 3. The prevalence of MetS was 27.9% in single-person households and 25.3% in multi-person households (*p* < 0.05). In addition, the prevalence of individual MetS components significantly differed by household type (all *p* < 0.05). Single-person households showed a higher prevalence of excessive WC (34.1%), elevated BP (38.0%), and elevated FBG (31.8%) than multi-person households (excessive WC: 31.3%; elevated BP: 32.4%; and elevated FBG: 28.4%; all, *p* < 0.01).

**Figure 4:**
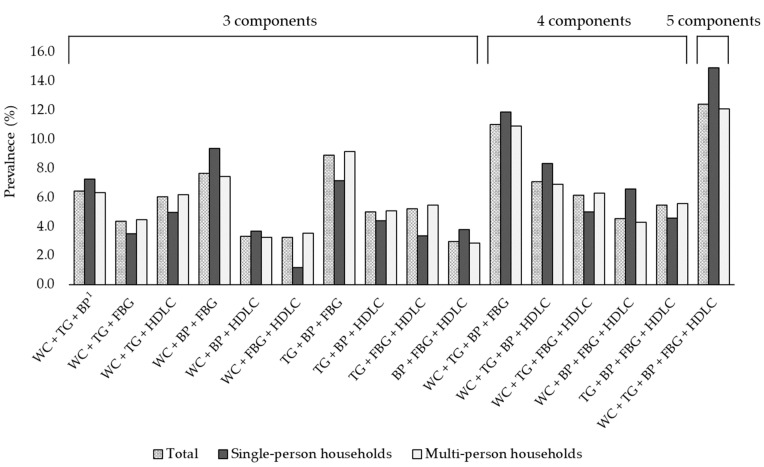
Prevalence of each metabolic syndrome component combination among Korean adults according to household type, KNHANES 2014–2018. KNHANES, Korea National Health and Nutrition Examination Survey; WC, waist circumference; TG, triglycerides; BP, blood pressure; FBG, fasting blood glucose; HDL-C, high-density lipoprotein cholesterol. ^1^ Each component of MetS was defined as follows: (1) excessive WC (WC ≥ 90 cm in men and ≥85 cm in women); (2) elevated TG (fasting TG ≥ 150 mg/dL or receiving treatment for hypertriglyceridemia); (3) elevated BP (systolic BP ≥ 130 mmHg or diastolic BP ≥ 85 mmHg or receiving treatment for or previously diagnosed with hypertension); (4) elevated FBG (FBG ≥ 100 mg/dL or receiving treatment for or previously diagnosed with type 2 diabetes mellitus); and (5) low HDL-C (fasting HDL-C < 40 mg/dL in men and < 50 mg/dL in women).

The differences in MetS component combinations according to household type are shown in Figure 4. Among single-person households, the most common combination of MetS components was the presence of all five components, followed by the WC + TG + BP + FBG, TG + BP + FBG, WC + BP + FBG, and WC + TG + BP + HDL-C combinations. The top five listed combinations for single-person households included excessive WC and elevated BP. The most common combinations in multi-person households also were the presence of all five components and the WC + TG + BP + FBG, followed by the WC + BP + FBG combination, followed by WC + TG + BP + HDL-C and WC + TG + BP combinations. Unlike for those living in single-person households, the top five ranked combinations for those living in multi-person households included elevated BP alone.

Among the variables described in Table 1, the factors associated with MetS according to household type are shown in Table 5. Overall, in single-person households, older age, lower education level, unemployment, and lack of regular physical activity were associated with higher odds of developing MetS (*p* < 0.05). In addition to the factors shown to be significantly related to MetS in single-person households, the following factors were also identified in multi-person households: older age, lower education and income levels, unemployment, drinking >1 time/month, and current smoking (all, *p* < 0.05).

The multivariable-adjusted ORs for MetS and its components according to household type are shown in Table 6. After controlling for potential covariates, including sex, age, education level, household income, marital status, occupation, region, drinking status, smoking status, and regular physical activity, individuals in single-person households showed higher odds of MetS (adjusted OR (AOR): 1.14, 95% CI: 1.02–1.29), abdominal obesity (AOR: 1.14, 95% CI: 1.01–1.28), elevated BP (AOR: 1.28, 95% CI: 1.12–1.47), and elevated FBG (AOR: 1.18, 95% CI: 1.05–1.33) than those living in multi-person households.

## 4. Discussion

This nationwide, population-based analysis of Korean adults showed that dietary behaviors and the prevalence of MetS differed by household type. Single-person households constituted approximately 9.2% of the study population. Compared with individuals in multi-person households, those in single-person households tended to be older, single, current smokers, and had lower education level and household incomes. The prevalence of MetS increases with age. This can be explained by the increased risk of metabolic diseases that comprise MetS, and the deficiency of sex hormones associated with increasing age [39,40]. In addition, compared with multi-person households, single-person households had lower levels of education and household income. Higher socioeconomic status has been reported to be the best predictor of good health, and among various socioeconomic factors, education level is strongly associated with MetS [41]. Since education level affects an individual’s ability to acquire and understand knowledge, those with a higher level of education can more easily access and utilize information that helps improve their health [42]. The Metabolic Syndrome Fact Sheet in Korea 2021 also reported that individuals with a lower education level and household income and those who are current smokers have a higher prevalence of MetS [13]. This indicates that the characteristics of single-person households found in our study overlapped with those of socioeconomic status and lifestyles reported as vulnerable to MetS in previous studies.

In our study, living alone was positively associated with the prevalence of MetS, excessive WC, elevated BP, and elevated FBG, independent of sex, age, education level, household income, marital status, occupation, region, drinking and smoking status, and regular physical activity. These findings are consistent with previous observations based on the analysis of the Korea Community Health Survey, which indicate that living alone is associated with greater morbidity, including higher rates of hypertension, diabetes, and dyslipidemia [43].

Though the percentage of energy from carbohydrates, proteins, and fats fell within the recommended AMDR irrespective of household type, we also found that single-person households had a higher total energy intake and percentage energy from animal-based foods, fats, and animal proteins and a lower energy intake from plant-based foods than multi-person households. The intake of animal-derived foods, fats, and animal proteins has been associated with metabolic abnormalities. In several prospective cohort studies, positive associations between the incidence of MetS and the percentage of energy intake from total and animal proteins have been found, while an inverse association has been reported between the incidence of MetS and its components and plant proteins [44,45,46]. In our study, individuals in single-person households had a higher energy intake from total and saturated fats than those in multi-person households; however, there were no differences in energy intake from polyunsaturated fats based on household type. A recent systematic review found that both the quality and quantity of dietary fat are associated with MetS and its individual components and that replacing saturated fats with mono- or polyunsaturated fats has a beneficial effect on cardiometabolic health [47]. Therefore, nutritional strategies targeting single-person households should encourage the choice of foods high in unsaturated fats rather than saturated fats.

Single- and multi-person households showed different food consumption patterns among Korean adults. Compared with multi-person households, those living in single-person households consumed more milk/dairy products, beverages, and oils/fats and fewer vegetables/mushrooms, fruits, seaweeds, and fish/shellfish. Consistent with our findings, previous studies have documented that individuals living in single-person households have fewer opportunities to purchase fruits, vegetables, and fish, making it difficult to consume a variety of foods [48,49]. Single-person households tend to consume refined grains and pickled vegetables, which are easier to store, rather than whole grains and raw vegetables [50,51]. Furthermore, those living with others consume a wider variety of food groups than those living alone. Frequent family meals are associated with higher consumption and availability of healthy foods, such as fruits and vegetables [52,53,54]. Choi et al. also reported that adults living in single-person households have a higher proportion of unhealthy eating habits, such as unbalanced and salty food consumption [55]. Single-person households eat alone relatively more frequently, and therefore, food choices and intake are likely to be biased based on their preferences.

Individuals living alone tend to have a more imbalanced nutrient intake, a lower quality diet, and tend to eat out more frequently than those living with others [48,49,50]. Accordingly, this study demonstrated that those living alone were more likely to eat meals out at least once per week and skip breakfast and were less likely to report consuming all main meals. Moreover, along with the increase in single-person households, the tendency to eat alone was noticeable, which was further exacerbated by the coronavirus disease pandemic. Consistent with this, surprisingly, the proportion of people who reported eating all their meals alone during the 24-h dietary recall day was approximately five times higher than that of multi-person households (22% in single-person households vs. 4% in multi-person households). Communication during meals with others increases appetite and leads to feelings of emotional support [56]. Therefore, eating with others, including family members, has been emphasized as a strategy to improve metabolic and mental health across the lifespan. In addition, we found that individuals living in single-person households experience more food insecurity than those in multi-person households. In our previous study of 685,327 Korean adults, we found a positive association between food insecurity and diabetes among single-person households regardless of where they lived [57]. Consistent with our findings, many studies have indicated that people living alone are more likely to encounter financial difficulties due to limited financial resources than those living with other family members [58,59]. Thus, strategic support for food security in single-person households is essential.

There are a few limitations to this study. First, owing to the cross-sectional nature of the study design, causal relationships could not be determined. Second, we used the dietary data obtained from a single 24-h dietary recall; however, this method may lead to random and systematic errors that produce bias and result in false information on the absolute intake of nutrients and foods. In addition, a single 24-h dietary recall may have limitations in capturing an individual’s usual intake. Though it is recommended that at least two days of 24-h dietary recall be used to estimate an individual’s usual intake, these data were not available. However, in our study, this limitation was overcome to some extent because we compared nutritional status and food consumption between single- and multi-person households rather than comparing the absolute intake of nutrients and foods by household type. Third, household types were defined based on the participants’ self-reports, which may differ from the types of households in which they reside. Recent changes, such as the increasing development of transportation and relocation of firms and public sectors to non-metropolitan areas, have resulted in a complex pattern of individual household types (for example, living alone during the week, and living and spending time with family members on weekends). These complex patterns of household types may lead to differences in dietary behaviors and health status, though detailed information about this was not available; thus, our study could not be extended to address this issue. Despite the limitations mentioned above, to the best of our knowledge, this is the first study to explore household types as an independent indicator of MetS and to investigate the relationships between household type and dietary behaviors and the prevalence of MetS in the general Korean population. The strengths of this study include the use of a nationally representative sample of Korean adults with a broad range of ages and regions. Furthermore, this study provides important preliminary findings on the role of household type as a predictor of MetS and its components. Additional investigations using prospective cohort studies are necessary to determine the longitudinal effects of household type on the development of MetS.

## 5. Conclusions

We found differences in nutrient intake, food consumption, dietary behaviors, and prevalence of MetS among Korean adults according to household type. Socioeconomic and health-related factors associated with MetS were shown to vary depending on the type of household. Considering that individuals living in single-person households were more likely to have health and dietary behaviors known to be risk factors for MetS than multi-person households, it is necessary to establish a differentiated health care system and nutrition policy based on household type.

## Figures and Tables

**Table 1:** General characteristics of study participants according to household types in Korean adults, KNHANES 2014–2018.

	Total	Household Type		*p*-Value ^1^
		Single-Person Households	Multi-Person Households	
	***n*** **(Wt’d %)**	***n*** **(Wt’d %)**	***n*** **(Wt’d %)**	
Total	21,944 (100.00)	2522 (9.19)	19,422 (90.81)	
Mean number of household members	3.09 ± 0.02 ^2^	1.00 ± 0.00	3.30 ± 0.01	
Sex				0.3528
Men	9143 (49.64)	945 (50.82)	8198 (49.53)	
Women	12,801 (50.36)	1577 (49.18)	11,224 (50.47)	
Age (years)				<0.0001
19–29	2498 (18.19)	239 (19.92)	2259 (18.01)	
30–49	7465 (39.29)	422 (26.38)	7043 (40.60)	
50–64	6371 (26.52)	616 (21.88)	5755 (27.00)	
≥65	5610 (16.00)	1245 (31.82)	4365 (14.39)	
Education level				<0.0001
≤Middle school	6990 (22.76)	1402 (38.22)	5588 (21.20)	
High school	7100 (36.13)	572 (28.70)	6528 (36.88)	
≥College	7854 (41.11)	548 (33.08)	7306 (41.92)	
Income				<0.0001
Lowest	4058 (14.21)	1279 (40.27)	2779 (11.57)	
Lower middle	5397 (23.44)	592 (23.19)	4805 (23.46)	
Upper middle	6105 (29.95)	361 (20.52)	5744 (30.90)	
Highest	6384 (32.41)	290 (16.03)	6094 (34.06)	
Marital status				<0.0001
Married	18,530 (76.71)	1864 (55.17)	16,666 (78.89)	
Single	3414 (23.29)	658 (44.83)	2756 (21.11)	
Occupation				0.0011
No	8775 (35.76)	1259 (39.76)	7516 (35.36)	
Yes	13,169 (64.24)	1263 (60.24)	11,906 (64.64)	
Region				0.0209
Urban	17,845 (86.10)	1924 (83.31)	15,921 (86.38)	
Rural	4099 (13.90)	598 (16.69)	3501 (13.62)	
Drinking status				0.3283
Never/rarely	10,309 (41.38)	1353 (42.23)	8956 (52.95)	
≤1 time/month	7017 (35.71)	677 (33.94)	6340 (19.81)	
>1 time/month	4618 (22.91)	492 (23.83)	4126 (27.24)	
Smoking status				<0.0001
Never	13,695 (57.86)	1551 (52.95)	12,144 (58.36)	
Former smoker	4647 (21.67)	461 (19.81)	4186 (21.86)	
Current smoker	3602 (20.47)	510 (27.24)	3092 (19.78)	
Regular physical activity ^3^				0.6259
Yes	9856 (46.66)	1082 (47.27)	8774 (46.60)	
No	12,088 (53.34)	1440 (52.73)	10,648 (53.40)	

KNHANES, Korea National Health and Nutrition Examination Survey; Wt’d %, weighted percentage. ^1^ *p*-Values obtained from the chi-square test to examine differences in the distribution of variables according to household type. ^2^ Mean ± standard error. ^3^ Regular physical activity was defined as walking ≥5 times a week for ≥30 min each time.

**Table 2:** Energy and macronutrient intake of Korean adults according to household type, KNHANES 2014–2018.

	Household Type		*p*-Value ^1^
	Single-Person Households	Multi-Person Households	
	**Mean ± SE**	**Mean ± SE**	
Total energy, kcal	2021 ± 23	1963 ± 13	0.0165
Plant sources ^2^, % of energy	81.70 ± 0.34	82.70 ± 0.19	0.2247
Animal sources ^2^, % of energy	18.30 ± 0.34	17.30 ± 0.19	0.0063
Carbohydrates, % of energy	64.83 ± 0.30	65.80 ± 0.17	0.0019
Proteins, % of energy	14.85 ± 0.13	14.73 ± 0.07	0.3662
Plant proteins ^2^, % of energy	7.62 ± 0.06	7.84 ± 0.04	0.0004
Animal proteins ^2^, % of energy	7.23 ± 0.15	6.88 ± 0.08	0.0244
Total fats, % of energy	20.32 ± 0.24	19.47 ± 0.14	0.0014
Saturated fats, % of energy	6.40 ± 0.10	6.05 ± 0.05	0.0008
Monounsaturated fats, % of energy	6.35 ± 0.11	6.06 ± 0.06	0.0126
Polyunsaturated fats, % of energy	5.26 ± 0.08	5.05 ± 0.04	0.0125

KNHANES, Korea National Health and Nutrition Examination Survey; SE, standard error. ^1^ *p*-Values obtained from the multiple regression models after adjusting for sex, age, education level, household income, marital status, occupation, region, drinking status, smoking status, and regular physical activity. ^2^ Grains and associated products, starchy vegetables, sugars/sweets, legumes, nuts/seeds, vegetables/mushrooms, fruits, seaweed, alcoholic/non-alcoholic beverages, oils (plant-based), and others (plant-based) were classified as plant sources, while foods derived from meat, eggs, fish/shellfish, milk/dairy products, oils/fats (animal-based), and others (animal-based) were classified as animal sources.

**Table 3:** Food group consumption and dietary variety among Korean adults according to household type, KNHANES 2014–2018.

	Household Type		*p*-Value ^1^
	Single-Person Households	Multi-Person Households	
	**Mean ± SE**	**Mean ± SE**	
Grains and associated products	285.96 ± 4.32	283.70 ± 2.55	0.6376
Starchy vegetables	38.56 ± 2.77	37.02 ± 1.54	0.5867
Sugar and sweets	10.33 ± 0.50	9.85 ± 0.29	0.3636
Legumes	40.11 ± 2.32	37.26 ± 1.27	0.2668
Nuts and seeds	6.42 ± 0.57	7.75 ± 0.50	0.0544
Vegetables and mushrooms	295.75 ± 4.88	310.76 ± 3.36	0.0064
Fruits	151.61 ± 6.35	168.33 ± 3.66	0.0160
Seaweed	18.05 ± 1.60	28.28 ± 1.51	<0.0001
Meat	109.36 ± 4.31	102.62 ± 2.72	0.1283
Eggs	27.54 ± 1.60	24.72 ± 0.76	0.0660
Fish and shellfish	86.80 ± 3.93	97.73 ± 2.71	0.0121
Milk and dairy products	95.01 ± 5.29	74.74 ± 2.25	0.0001
Alcoholic and non-alcoholic beverages	356.67 ± 12.89	326.45 ± 7.31	0.0217
Oils and fats	7.93 ± 0.34	6.72 ± 0.15	0.0003
Others	37.30 ± 1.26	34.13 ± 0.67	0.0170
Total DVS	10.10 ± 0.07	10.43 ± 0.04	<0.0001

KNHANES, Korea National Health and Nutrition Examination Survey; SE, standard error; DVS, dietary variety score. ^1^ *p*-Values obtained from the multiple regression models after adjusting for sex, age, education level, household income, marital status, occupation, region, drinking status, smoking status, and regular physical activity.

**Table 4:** Dietary behaviors among Korean adults according to household type based on 24-h dietary recall, KNHANES 2014–2018.

	Household Types		*p*-Value ^1^
	Single-Person Households	Multi-Person Households	
	**Mean ± SE**	**Mean ± SE**	
Total eating episodes	5.32 ± 0.05	5.37 ± 0.03	0.3192
Total main meal episodes	2.54 ± 0.01	2.58 ± 0.01	0.0099
Total snacking episodes	2.77 ± 0.05	2.79 ± 0.03	0.7960
Energy from foods prepared at home, %	37.09 ± 0.73	46.06 ± 0.46	<0.0001
Energy from foods prepared outside the home, %	47.60 ± 1.17	43.35 ± 0.92	<0.0001
Report skipping breakfast, %	21.81 ± 1.10	17.09 ± 0.42	<0.0001
Report eating all three main meals, %	65.59 ± 1.30	70.30 ± 0.49	0.0007
Report eating all main meals alone, %	21.88 ± 1.08	4.00 ± 0.20	<0.0001
Report eating out at least 1 time per week, %	29.02 ± 1.66	18.05 ± 0.46	<0.0001
Mild/moderate or severe food insecurity, %	48.10 ± 1.39	44.65 ± 0.67	0.0202

KNHANES, Korea National Health and Nutrition Examination Survey; SE, standard error. ^1^ *p*-Values obtained from the multiple regression models after adjusting for sex, age, education level, household income, marital status, occupation, region, drinking status, smoking status, and regular physical activity.

**Table 5:** Factors associated with metabolic syndrome among Korean adults according to household type, KNHANES 2014–2018.

	Household Type	
	Single-Person Households	Multi-Person Households
	**AOR (95% CI) ^1^**	**AOR (95% CI)**
Sex		
Men	0.87 (0.65–1.17)	0.67 (0.60–0.75)
Women	1.00 (ref.)	1.00 (ref.)
Age (years)		
19–29	1.00 (ref.)	1.00 (ref.)
30–49	3.48 (2.09–5.80)	3.89 (3.02–5.00)
50–64	6.10 (3.40–10.94)	7.15 (5.50–9.29)
≥65	8.17 (4.46–14.97)	10.79 (8.18–14.22)
Education level		
≤Middle school	1.53 (1.08–2.17)	1.78 (1.58–2.01)
High school	1.15 (0.81–1.63)	1.26 (1.14–1.39)
≥College	1.00 (ref.)	1.00 (ref.)
Income		
Lowest	1.23 (0.81–1.89)	1.29 (1.12–1.48)
Lower middle	1.06 (0.68–1.65)	1.11 (1.00–1.24)
Upper middle	0.76 (0.50–1.15)	1.08 (0.97–1.20)
Highest	1.00 (ref.)	1.00 (ref.)
Marital status		
Married	1.00 (ref.)	1.00 (ref.)
Single	1.03 (0.68–1.56)	1.01 (0.83–1.25)
Occupation		
Unemployed	1.31 (1.04–1.65)	1.09 (1.01–1.19)
Employed	1.00 (ref.)	1.00 (ref.)
Region		
Urban	1.01 (0.79–1.30)	1.07 (0.94–1.20)
Rural	1.00 (ref.)	1.00 (ref.)
Drinking status		
Never/rarely	1.00 (ref.)	1.00 (ref.)
≤1 time/month	0.77 (0.60–1.01)	0.93 (0.85–1.02)
>1 time/month	0.83 (0.60–1.14)	1.20 (1.08–1.34)
Smoking status		
Never	1.00 (ref.)	1.00 (ref.)
Former smoker	0.80 (0.57–1.13)	1.00 (0.88–1.13)
Current smoker	1.13 (0.77–1.66)	1.24 (1.08–1.42)
Regular physical activity ^2^		
Yes	1.00 (ref.)	1.00 (ref.)
No	1.29 (1.04–1.60)	1.05 (0.97–1.13)

KNHANES, Korea National Health and Nutrition Examination Survey; AOR, adjusted odds ratio; 95% CI, 95% confidence interval. ^1^ Multiple logistic regression analysis was performed to estimate the odds for MetS for the study participants from the KNHANES 2014–2018. The statistical model was adjusted for sex, age, education level, household income, marital status, occupation, region, drinking status, smoking status, and regular physical activity, where applicable. ^2^ Regular physical activity was defined as walking ≥5 times a week for ≥30 min each time.

**Table 6:** Adjusted odds ratios with 95% confidence intervals for metabolic abnormalities among Korean adults according to household type, KNHANES 2014–2018.

	Household Type		*p*-Value
	Multi-Person Households	Single-Person Households	
		**AOR (95% CI) ^1^**	
MetS ^2^	1.00 (ref.)	1.14 (1.02–1.29)	0.0244 * ^3^
Excessive WC	1.00 (ref.)	1.14 (1.01–1.28)	0.0279 *
Elevated TG	1.00 (ref.)	1.09 (0.97–1.22)	0.1684
Elevated BP	1.00 (ref.)	1.28 (1.12–1.47)	0.0003 **
Elevated FBG	1.00 (ref.)	1.18 (1.05–1.33)	0.0072 **
Low HDL-C	1.00 (ref.)	1.04 (0.92–1.17)	0.5177

KNHANES, Korea National Health and Nutrition Examination Survey; AOR, adjusted odds ratio; 95% CI, 95% confidence interval; MetS, metabolic syndrome; WC, waist circumference; TG, triglycerides; BP, blood pressure; FBG, fasting blood glucose; HDL-C, high-density lipoprotein cholesterol. ^1^ Multiple logistic regression analysis was performed to estimate the odds of MetS and its components for the study participants from the KNHANES 2014–2018. The statistical model was adjusted for sex, age, education level, household income, marital status, occupation, region, drinking status, smoking status, and regular physical activity. ^2^ MetS was defined as the presence of at least three of the following conditions: (1) excessive WC, (2) elevated TG, (3) elevated BP, (4) elevated FBG, and (5) low HDL-C. ^3^ *p*-Values obtained from the multivariable logistic regression models with MetS and its components as the outcome variables (* *p* < 0.05, ** *p* < 0.01).

## Data Availability

The Korea National Health and Nutrition Examination Survey (KNHANES) 2014–2018 data used in this study can be found at the following link: https://knhanes.kdca.go.kr/knhanes/sub03/sub03_02_05.do (accessed on 1 October 2020).
